# Effects of past and present habitat on the gut microbiota of a wild rodent

**DOI:** 10.1098/rspb.2023.2531

**Published:** 2024-02-07

**Authors:** Tiffany Scholier, Anton Lavrinienko, Eva R. Kallio, Phillip C. Watts, Tapio Mappes

**Affiliations:** ^1^ Department of Biological and Environmental Science, University of Jyväskylä, Jyväskylä 40014, Finland; ^2^ Laboratory of Food Systems Biotechnology, Institute of Food, Nutrition and Health, ETH Zürich, Zürich 8092, Switzerland

**Keywords:** reciprocal transfer experiment, gut microbiota, translocation, flexibility, resistance, urban

## Abstract

The response of the gut microbiota to changes in the host environment can be influenced by both the host's past and present habitats. To quantify their contributions for two different life stages, we studied the gut microbiota of wild bank voles (*Clethrionomys glareolus*) by performing a reciprocal transfer experiment with adults and their newborn offspring between urban and rural forests in a boreal ecosystem. Here, we show that the post-transfer gut microbiota in adults did not shift to resemble the post-transfer gut microbiota of animals ‘native’ to the present habitat. Instead, their gut microbiota appear to be structured by both their past and present habitat, with some features of the adult gut microbiota still determined by the past living environment (e.g. alpha diversity, compositional turnover). By contrast, we did not find evidence of the maternal past habitat (maternal effects) affecting the post-transfer gut microbiota of the juvenile offspring, and only a weak effect of the present habitat. Our results show that both the contemporary living environment and the past environment of the host organism can structure the gut microbiota communities, especially in adult individuals. These data are relevant for decision-making in the field of conservation and wildlife translocations.

## 1. Introduction

Gut microbiota provide essential functions to their animal host, most notably by metabolizing dietary items to yield diverse nutrients [1] but also by interacting with the host's immune system to support the protection against pathogens [2]. The gut microbiota develops after birth to reflect the experiences of the host during early life and thereafter generally stabilizes with age [3–5]. Some of the most prominent environmental drivers of variation in wildlife gut microbiota include dietary input [6,7], parasite or pathogen burden [8,9], and exposure to pollution or habitat disturbance [10,11]. Moreover, maternal transmission [12] and social contact [13] can also impact the gut microbiota composition, although the influence of the maternal microbiota apparently weakens with age [12,14]. Thus, a typical feature of the animal gut microbiota is that conspecifics have distinct communities depending on where they live [11,15]. When an animal moves to a new environment, it is reasonable to expect a concomitant change in the gut microbiota, for example as a response to new resources [16–18] or contact with new conspecifics and/or pathogens. While the gut microbiota are key to animal health [19], how the gut microbiota respond to environmental change that accompany wildlife movement to new areas remains underexplored.

Following movement to a new area, the host's microbiota could exhibit either *flexibility* or *resistance* in response to any change in the environment. Flexible gut microbiota are hypothesized to enable the hosts to better use available resources [20–22]. Such flexibility may be achieved by the uptake of new microbes and/or by altering the composition of resident taxa. In both cases, the microbiota composition would reflect the contemporary (present) environment of the host. Alternatively, the gut microbiota can reflect the host's prior environmental experiences by exhibiting *resistance*. Mechanisms of microbiota *resistance* may be derived from the microbial communities that resist a change in composition through priority effects [23–25], whereby the early-arriving (and thus established) taxa inhibit or influence the colonization success of later-arriving microbes [26–28]. Also, the host could stimulate *resistance* by providing a stable environment (e.g. host mucus [29]). Studying the relative contributions of *flexibility* and *resistance* in terms of animal movement is important, considering the ongoing fragmentation of wild habitats and the practice of human-assisted wildlife translocations for conservation purposes [30].

Few studies have quantified the relative influence of *flexibility* and *resistance* in gut microbiota of wild animals, likely owing to the requirement of longitudinal data from a reciprocal transfer (RT) experiment. Nonetheless, there is some evidence for gut microbiota *flexibility* [31] and an interaction whereby the host's prior environment may determine the degree of *flexibility* [32,33]. The apparent absence of a notable effect of prior experience on the microbiota composition in these RT studies may be explained by their focus on invertebrates [33] or juvenile life stages of aquatic vertebrates [31,32]. Indeed, invertebrates typically harbour a much less complex microbiota community than vertebrate hosts, and often incorporate a greater proportion of transient, free-living microbes in their gut microbiota [34]. Similarly, the responses of gut microbiota of juveniles may not be comparable with those of adults owing to the differences in the gut microbiota maturity and relative importance of priority effects [25], early-life conditions [35,36] and maternal effects [14]. Therefore, RT experiments performed on both adults and juveniles are needed to elucidate the importance of past and present habitat in different age classes.

Importantly, mothers may vertically transfer (part of) their gut microbiota to their offspring [12,14,37]. In mammals, this process of vertical transmission is stimulated by birth through the vaginal canal, breast feeding, coprophagy of maternal stool, and close physical contact between mother and offspring [38]. With this in mind, conducting an RT experiment on mothers and their newborn offspring, which have not directly experienced the maternal prior environment, would provide novel insights into the combined roles of maternal transmission and gut microbiota *flexibility* in shaping variation in the gut microbiota. Under a predominant process of microbiota *flexibility*, an individual's contemporary environment would have the best explanatory power of gut microbiota composition (e.g. as gut microbiota is primarily influenced by diet [6,7]). However, if there is *resistance*, whereby the prior environmental experience continues to impact the maternal gut microbiota after translocation, then the ‘legacy’ of the maternal prior environment may be visible in the offspring owing to maternal transmission. Whether the gut microbial communities in offspring reflect the prior maternal environment (before maternal movement) has, to the best of our knowledge, not been studied using an RT experiment.

In this study, we quantified the contributions of prior (i.e. past habitat) and contemporary environments (i.e. present habitat) on the gut microbiota composition by performing an RT experiment with adult wild rodents and their newborn offspring (the bank vole, *Clethrionomys glareolus*; formerly *Myodes glareolus*; [39]) inhabiting contrasting (urban and rural) forest habitats. We chose this model system since both environmental [40] and host-associated [15,41] microbiota differ between urban and rural forests, thus allowing us to detect *flexibility* and *resistance* after translocation. In line with previous RT studies [31–33], we hypothesized that (1) the adult post-transfer gut microbiota will be explained more by the contemporary environment than by the prior environment. Additionally, we anticipated that (2) the gut microbiota of offspring will solely be determined by the contemporary environment (i.e. with a negligible signal of the maternal prior environment). Thus, we expected an overriding signature of gut microbiota *flexibility* rather than *resistance*, especially in juveniles.

## 2. Methods

### (a) Experimental design

**Figure 1.**
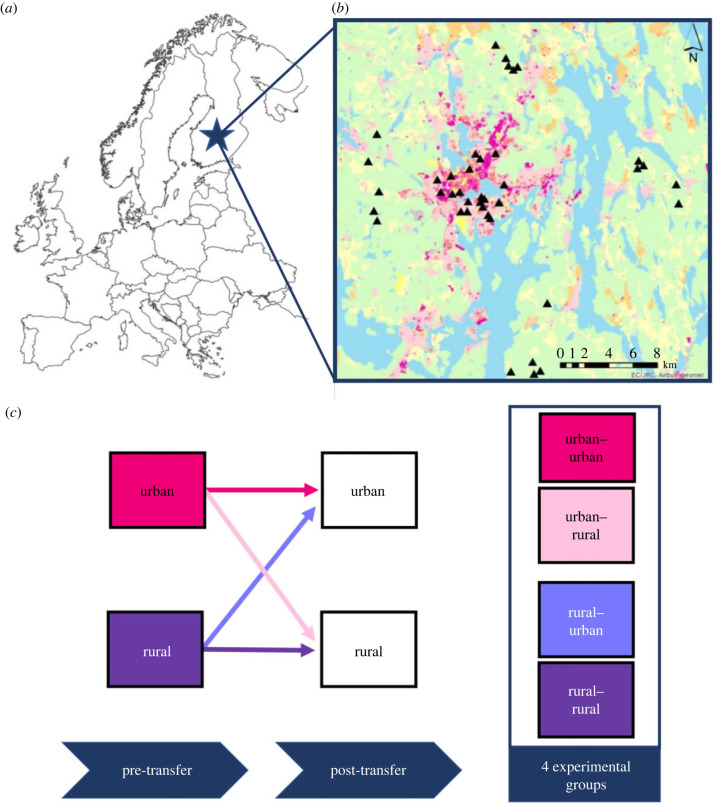
Experimental design of the reciprocal transfer (RT) experiment on wild bank voles. The experiment was carried out in Jyväskylä, situated in central Finland (*a*) and used 20 urban and 20 rural forest sites around the city (pink areas represent urban zones) as experimental forest patches (*b*). The schematic overview of the reciprocal transfer experiment shows that wild gravid bank voles were sourced from urban and rural forests (referred to as 'past habitat') and released in a new urban or rural forest patch (referred to as 'present habitat') with their newborn offspring (*c*). Data from pre-transfer gut microbiota are based on faecal samples derived from bank voles upon initial capture, while the post-transfer microbiota were obtained from animals after spending four weeks in their new forest patch after translocation.

Nursing bank voles were transferred according to a full factorial reciprocal transfer (RT) design between 20 urban and 20 rural forest sites within and around Jyväskylä (62.2426°N, 25.7473°E) in central Finland, during July–September 2020. Rural sites were established around the urban area and were subdivided into four areas with five forest sites each to avoid potential biases associated with any specific location. The typical vegetation found in urban and rural forest sites was comparable, comprising the same tree (Norway spruce (*Picea abies*), Scots pine (*Pinus sylvestris*), and silver and downy birch (*Betula pendula* and *Betula pubescens*)) and shrub species (bilberry (*Vaccinium myrtillus*) and lingonberry (*Vaccinium vitis-idaea*)). All 40 sites were used as animal sourcing and release plots. To supplement the numbers of rural bank voles, animals were also sourced from rural forests located 50 km away from Jyväskylä. The experiment consisted of three phases (figure 1): (1) sourcing gravid bank voles from the wild and sampling the original faecal microbiota (i.e. pre-transfer microbiota), (2) keeping these individuals in the laboratory until 2–3 days after giving birth (for recovery; time period until birth varied per individual: 3–20 days), after which animals were transferred with their offspring to a new forest patch (animals were never transferred to their original forest patch, such that all animals had the same experience of moving to a new area), and (3) recapturing the same individuals and their pups and sampling their faecal microbiota after having spent 3–4 weeks in the wild (i.e. post-transfer microbiota). This effort resulted in data for 28 adult bank voles (rural–rural *n* = 7; rural–urban *n* = 5; urban–rural *n* = 7; urban–urban *n* = 9) that had both pre- and post-transfer microbiota samples and 72 offspring that had a post-transfer microbiota sample (rural–rural *n* = 17; rural–urban *n* = 26; urban–rural *n* = 10; urban–urban *n* = 19). Recaptured juveniles belonged to 33 mothers (note that the mother was not always recaptured and, in some cases, several siblings were recaptured). We used nursing mothers to increase the recapture rate since nursing individuals are less likely to disperse. Full details about the experimental design and sampling protocols can be found in the methods section of the electronic supplementary material.

### (b) Amplicon sequencing and read data processing

DNA from faecal samples (total *n* = 128) and negative controls was extracted in a randomized order at the facilities of the University of Jyväskylä (JYU) using a Qiagen DNeasy PowerSoil Pro Kit following the manufacturer's instructions. The DNA concentrations per sample (see electronic supplementary material, methods) were measured with a Nanodrop spectrophotometer at JYU and confirmed with a Qubit fluorometer at the Beijing Genomics Institute (BGI, www.bgi.com/global/). Amplification and sequencing of the DNA samples were performed at BGI using an Illumina HiSeq with the primer pair 515F/806R [42] to target the V4 region of the 16S ribosomal RNA (rRNA) locus. To enhance quality control, negative controls were also incorporated during the library preparation step. The following workflow was used in qiime2 v.2021.8 [43] to attain amplicon sequence variants (ASVs): removal of adaptor sequences with the cutadapt plugin [44], trimming primers, truncating the 3′ end of the low-quality reads (forward reads at 227 bp, reverse reads at 199 bp), merging paired reads, and filtering out chimeric sequences with the dada2 plugin [45]. Taxonomy was assigned to ASVs by training the Naive Bayes classifier [46] on the SILVA database v.13_8 for the V4 region of 16S rRNA [47], with reference sequences clustered at 99% sequence similarity. After removal of non-bacterial (Archaea, Eukaryota, mitochondria, chloroplasts, and unassigned sequences) and low-frequency ASVs (i.e. <10 reads overall), 10 432 395 reads (30 671–137 472 reads/sample) and 3448 ASVs were retained, with the final amount of 3 925 888 reads and 3443 ASVs after rarefaction at 30 671 reads per sample [48]. Phylogenetic midpoint rooted trees were constructed using the fasttree plugin [49].

### (c) Statistical analysis

#### (i) Magnitude of change in alpha and beta diversity

The analysis of our paired time series data for adults was performed with the q2-longitudinal plugin [50] in qiime2. For each adult bank vole, we calculated the difference in alpha diversity (paired-differences) and the change in beta diversity (paired-distances) between the two timepoints (i.e. the pre-transfer and post-transfer gut microbiota). As this approach generates independent response variables, we ran linear regression models with the *lm* function in R v.4.0.2 [51] to determine whether the magnitude of change in alpha (i.e. ASV richness, Shannon diversity, Faith's phylogenetic diversity) and beta diversity (i.e. Bray–Curtis, Jaccard, weighted UniFrac (wUnifrac), unweighted UniFrac (unwUnifrac)) depended on the past habitat, present habitat and/or their interaction. Here, the past and present habitat refer to a categorical variable describing the original and transfer forest type (either urban or rural). Additionally, we investigated whether the change in alpha diversity within treatment was statistically different from zero with two-sided paired *t*-tests with the *t_test* function in R.

#### (ii) Direction of change in alpha and beta diversity

To determine whether potential changes in alpha and beta diversity were deterministic or stochastic, we studied the post-transfer gut microbiota communities of adult bank voles in more detail. For alpha diversity analysis, we extracted the different alpha diversity metrics from qiime2 (i.e. ASV richness, Shannon diversity and Faith's phylogenetic diversity) and used the *lm* function in R to construct linear regression models with the two categorical variables of interest (i.e. past and present habitat) and their two-way interaction to examine potential cross-effects before and after the transfer. Linear models were also used to test whether the past habitat determined the alpha diversity of the pre-transfer gut microbiota.

In terms of beta diversity, distance metrics (i.e. Bray–Curtis, Jaccard, wUnifrac, unwUnifrac) were generated with the phyloseq v.1.44.0 package in R [52] and PERMANOVA tests were performed using the *adonis2* function in vegan v.2.6.4 (permutations *n* = 9999 [53]) to examine whether the past habitat, present habitat and/or their interaction had more influence on the composition of the post-transfer gut microbiota communities. The same function was used to study the effect of the past habitat on the composition of the pre-transfer gut microbiota. Additionally, the *betadisper* function in vegan with an adjustment for the potential bias in sample sizes was used to test for the assumption of homoscedasticity.

To visualize the directionality of the change in beta diversity, we used constrained analysis of principal coordinates (CAP) plots which display the variation explained by the *a priori* given hypothesis (i.e. past habitat + present habitat) with the *ordinate* function within phyloseq.

#### (iii) Alpha and beta diversity metrics in offspring

Similar analyses were applied to the post-transfer gut microbiota of the juveniles (*n* = 72) but with the relatedness between siblings (i.e. siblings were merged into clusters, cluster *n* = 33) taken into account. To study the alpha diversity between groups, we constructed linear mixed models with the alpha diversity index (i.e. ASV richness, Shannon index, Faith's phylogenetic diversity) as the response variable, the two-way interaction between past and present habitat as explanatory variables and genetic cluster as the random effect with the *lmer* function within the lmertest v.3.1.3 R package [54]. When needed, the non-normal distribution of the response variable was solved by log-transformation. In terms of investigating beta diversity differences, we modelled a for-loop function (iterations *n* = 100) where each loop started with the random selection of one pup per genetic cluster (*n* = 33) before running the *adonis2* function in R (permutations *n* = 9999). The average of the calculated values was taken as a representative for the whole dataset of offspring.

## 3. Results

### (a) Baseline differences in the bank vole gut microbiota

Throughout the experiment, the gut microbiota of adult bank voles was approximately 80% dominated by three bacterial families (electronic supplementary material, figure S1A): *Muribaculaceae* (median relative abundance pre- and post-transfer gut microbiota combined = 50.17%), *Lactobacillaceae* (median relative abundance pre- and post-transfer gut microbiota combined = 24.22%) and *Lachnospiraceae* (median relative abundance pre- and post-transfer gut microbiota combined = 10.17%).

The composition of the pre-transfer gut microbiota differed between wild urban and rural bank voles. Specifically, the community composition of bank vole gut microbiota differed among the two habitat types (*p* < 0.05 for all metrics except wUnifrac metric; electronic supplementary material, table S1 and figure S2), with habitat type explaining 7–9% of variation, depending on the metric used. The level of dispersion in the pre-transfer gut microbiota composition between urban and rural animals was similar based on three out of four beta diversity metrics used, though rural animals exhibited higher dispersion based on the unwUnifrac metric (*p* < 0.05; electronic supplementary material, table S1). By contrast, initial alpha diversity levels of the pre-transfer gut microbiota did not differ between rodents of different origin, with urban and rural bank voles having an average of 267 and 265 ASVs, respectively (electronic supplementary material, table S2). These differences in the pre-transfer gut microbiota profiles between urban and rural bank voles confirm the prerequisite needed to use this system for an RT experiment.

### (b) Past habitat determines the diversity and compositional turnover of the gut microbiota in translocated adults

**Figure 2.**
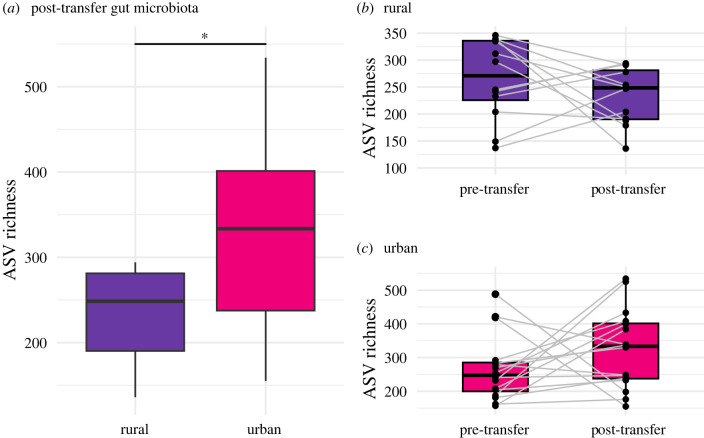
Change in the alpha diversity of the gut microbiota of bank voles in terms of past habitat. The alpha diversity (ASV richness) of the post-transfer gut microbiota was significantly higher for animals that originated from urban forests in comparison with rural forests, irrespective of their site of translocation (*a*). Connected bar plots show the increase or decrease in alpha diversity in the gut microbiota (paired-differences in alpha diversity) for each individual bank vole between their pre-transfer and post-transfer gut microbiota. Bank voles originating from rural (*b*) and urban (*c*) forests are shown in separate plots. The asterisk corresponds to a significant *p*-value (*p < 0.05).

Change in alpha diversity between the pre- and post-transfer microbiota was not statistically different between bank voles of different experimental groups (*p* > 0.05; electronic supplementary material, figure S3A and tables S3 and S4), and yet the alpha diversity of post-transfer microbiota was statistically different based on the past habitat (figure 2 and electronic supplementary material, table S5). Animals of urban origin had on average 30% more ASVs (*p* < 0.05) in their post-transfer microbiota communities (average of 324 ASVs) compared with rural bank voles (average of 234 ASVs), irrespective of their present habitat (ASV richness: $R_{\mathrm{past}}^{2}=0.16,\;p_{\mathrm{past}}=0.02$; figure 2 and electronic supplementary material, figure S3B and table S5). A trend towards higher alpha diversity in the post-transfer microbiota was also observed in terms of the Shannon diversity ($R_{\mathrm{past}}^{2}=0.102,\;p_{\mathrm{past}}=0.054$) and the Faith's phylogenetic diversity ($R_{\mathrm{past}}^{2}=0.063,\;p_{\mathrm{past}}=0.105$), although these results were non-significant (electronic supplementary material, table S5). In terms of beta diversity, all bank voles exhibited baseline temporal changes in the composition of their gut microbiota (i.e. change in their microbiota communities between the onset and end of the RT experiment), irrespective of whether they were transferred to similar forests (urban–urban, rural–rural) or to forests of the opposite experimental type (urban–rural, rural–urban) (electronic supplementary material, figure S4 and table S6), which likely reflects broad seasonal trends. Interestingly, these microbiota turnover rates were significantly higher for animals originating from urban rather than rural forests in terms of the wUniFrac metric ($R_{\mathrm{past}}^{2}=0.187,\;p_{\mathrm{past}}=0.013$; electronic supplementary material, figure S4 and table S6) but not for the other metrics. Additionally, no obvious trends in terms of the present habitat could be detected.

### (c) Past and present habitat determine the composition of the gut microbiota in translocated adults

**Figure 3.**
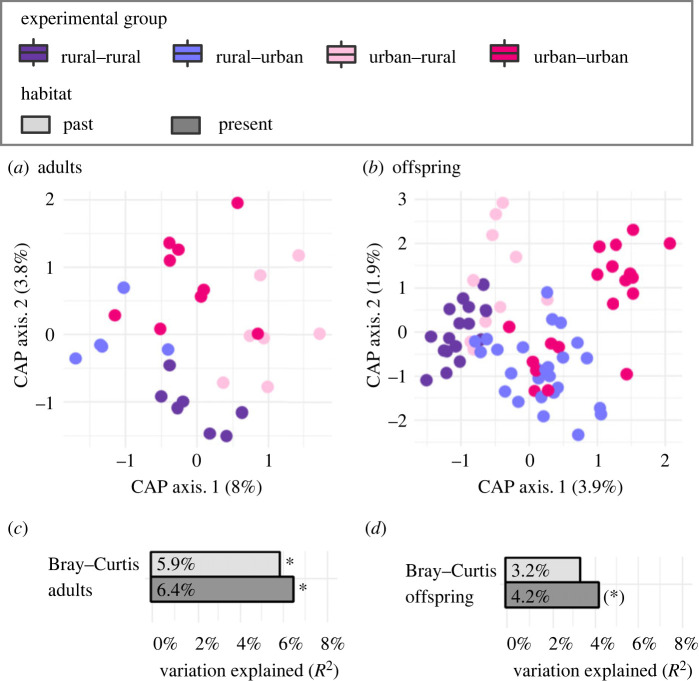
Post-transfer gut microbiota compositions of bank voles in terms of past and present habitat. The ordination plots for adults (*a*) and juvenile offspring (*b*) are based upon the Bray–Curtis metric while the *R*^2^ values and statistical significance values for adults (*c*) and juvenile offspring (*d*) are based upon the output from *adonis2* tests. Overall, the plots show that clustering of the adult post-transfer gut microbiota occurs according to the past and present habitat (*p* < 0.05) while the juvenile post-transfer gut microbiota has no significant influence from either. However, there is a trend towards clustering according to the present habitat. The asterisk corresponds to a significant *p*-value (*p < 0.05). CAP, constrained analysis of principal coordinates.

The effect sizes of the past and present habitat in terms of their contribution to the variation in the post-transfer gut microbiota were equivalent (approx. 5–6%; figure 3*a,c* and electronic supplementary material, figure S5 and table S7). Specifically, we found significant effects for both the past and present habitat in terms of the Bray–Curtis metric ($R_{\mathrm{past}}^{2}=0.059,\;F_{\mathrm{past}}=1.685,$ $p_{\mathrm{past}}=0.029;\;R_{\mathrm{present}}^{2}=0.065,\;F_{\mathrm{present}}=1.832,\;p_{\mathrm{present}}=0.016$) and the Jaccard metric ($R_{\mathrm{past}}^{2}=0.05,\;F_{\mathrm{past}}=1.381,\;\;p_{\mathrm{past}}=$ $0.038;\;R_{\mathrm{present}}^{2}=0.055,\;F_{\mathrm{present}}=1.527,$ $p_{\mathrm{present}}=0.013$), a significant effect of the past habitat in terms of the unwUnifrac metric ($R_{\mathrm{past}}^{2}=0.048,$ $F_{\mathrm{past}}=1.297,\;p_{\mathrm{past}}=0.044$) and no clustering trends in terms of the wUnifrac metric (*p* > 0.05). Additionally, we found that the past habitat had an influence on the dispersion levels in the post-transfer gut microbiota (electronic supplementary material, table S7), with the gut microbiota of animals originating from urban forests displaying greater levels of dispersion for all metrics (*p* < 0.02) except the unwUnifrac metric (*p* > 0.05).

### (d) Present habitat shapes the gut microbiota in translocated offspring

Gut microbiota of juvenile bank voles were dominated by the same three bacterial families as the gut microbiota of adults (electronic supplementary material, figure S1B), with *Muribaculaceae* (median relative abundance = 53.35%), *Lactobacillaceae* (median relative abundance = 21.8%) and *Lachnospiraceae* (median relative abundance = 9.14%) together constituting an average of 80% of the gut microbiota composition.

As expected, we found that both the alpha ($R_{\mathrm{geneticcluster}}^{2}\approx$ $0.32\text{–}0.42,\;p_{\mathrm{geneticcluster}}<0.02$ for all metrics) and beta diversity ($R_{\mathrm{geneticcluster}}^{2}\approx 0.61\text{–}0.68,\;p_{\mathrm{geneticcluster}}=0.0001$ for all metrics) of the juvenile gut microbiota were heavily influenced by the genetic cluster, with siblings having a higher similarity of the gut microbiota as compared with unrelated individuals (electronic supplementary material, figure S6 and table S8).

While the gut microbiota composition of translocated adults was influenced by both their past and present habitats, the microbiota of their offspring did not display a clear signature from the maternal past habitat. Instead, the gut microbiota of the offspring appeared to align according to their present habitat as assessed by the Bray–Curtis ($R_{\mathrm{past}}^{2}=$ $0.032,\;F_{\mathrm{past}}=1.041,\;p_{\mathrm{past}}=0.391;\;R_{\mathrm{present}}^{2}=0.042,\;F_{\mathrm{present}}=$ $1.362,\;p_{\mathrm{present}}=0.061$) and Jaccard metrics ($R_{\mathrm{past}}^{2}=0.032,$ $F_{\mathrm{past}}=1.041,\;p_{\mathrm{past}}=0.378;\;R_{\mathrm{present}}^{2}=0.038,\;F_{\mathrm{present}}=1.22,$ $p_{\mathrm{present}}=0.061$), although the results did not achieve statistical significance (figure 3*b*,*d*; electronic supplementary material, figure S5 and table S8).

## 4. Discussion

The composition of the wildlife gut microbiota typically exhibits a high level of inter-individual variation, likely driven by differential maternal effects and accumulated effects of diverse socio-environmental experiences throughout the host's lifetime. By performing a reciprocal transfer (RT) experiment with wild bank voles inhabiting urban and rural forests, we show that the past and present habitat have comparable explanatory power in terms of the variation in the post-transfer adult gut microbiota. Finding evidence for both *resistance* and *flexibility* is in contrast with our first hypothesis, as we expected that the gut microbiota *flexibility* would override any remaining signatures of the host's past habitat. That said, our results generally align with our second hypothesis, as indeed we expected to find little notable impact of the maternal past habitat on the gut microbiota of translocated offspring (i.e. juveniles), but rather an effect of the present habitat. Specifically, our data show that both past and present habitat do not significantly explain the variation in the juvenile gut microbiota, with only a trend suggesting an impact of the present habitat. Overall, our data highlight that wild gut microbiota studies should consider the effects of both the past and present habitats encountered in an individual's lifetime, while potential legacy effects of the maternal past habitat are relatively small.

### (a) Past habitat can maintain long-term differences in the adult bank vole gut microbiota

The past habitat rather than the present habitat determined many aspects of the adult bank vole gut microbiota response. Greater levels of compositional turnover, higher post-transfer alpha diversity and higher levels of dispersion in the post-transfer gut microbiota of animals originating from urban forests could be due to differences on the level of the microbiota and/or the level of the host organism. For example, it is possible that urban-specific gut microbiota harbour more transient microbes. Alternatively, urban animals could have different physiology and/or immune system features [55,56] that might allow a higher passive uptake of environmental microbes [57]. It also cannot be excluded that urban animals experienced more stress throughout the RT experiment and therefore show signs of ‘disturbance’, which, for example, could be consistent with the Anna Karenina principle for animal microbiomes, which postulates that stress increases the dispersion of the gut microbiota composition [58]. Additionally, urban animals may exhibit more bold and exploratory behaviour, thus encountering a greater variety of microhabitats and dietary items [59]. Intriguingly, such differences in the gut microbiota response between urban and rural bank voles can also be attributed to other host features such as genetic variation [41] and/or early life adaptations [23,24] tailored specifically to life in urban and rural forests. For example, an urban lifestyle during the critical period of early development could have a different impact on the maturation of the immune and stress response systems of bank voles in comparison with life in rural forests [60]. Consequently, such potential differences in developmental trajectories and, for instance, the close interplay between the immune system and the gut microbiota [61] could cascade to differences in the gut microbiota communities of adult animals. Moreover, it is also possible that changes in genetic structure between urban and rural bank voles have developed over time on the population level, owing to the geographical isolation of forest habitats.

### (b) Flexibility and resistance rather than maternal effects shape the bank vole gut microbiota

Both the past and present habitat shape the gut microbiota of translocated adult individuals, with each explaining between 5 and 6% of variation, depending on the metric used. While the capability of the gut microbiota to resemble the present habitat after a change in the host environment was expected [31–33], it is interesting to find a persistent signature of the past habitat in the post-transfer gut microbiota given the animals' time in captivity before translocation and their extended exposure to the new environment. Specifically, we found that the post-transfer microbiota of bank voles transferred between different forest types (urban–rural, rural–urban) did not match the ‘native’ microbiota of the transfer environment (urban–urban, rural–rural), but rather reached intermediate states. Temporal variation in the gut microbiota composition, irrespective of the forest type, is an important feature of our longitudinal data, and is consistent with seasonal changes in gut microbiota of wild rodents [62]. With this in mind, and the significant differences in the gut microbiota among urban and rural bank voles (electronic supplementary material, table S1), we had expected a considerably stronger effect size for *flexibility*. The inability of the gut microbiota communities of bank voles to be fully flexible could indicate the presence of strong priority effects that drive the gut microbiota *resistance* [32].

The influence of past environments on the gut microbiota communities appears to be limited to exposures faced over the course of an individual's lifetime, without a cross-generational effect, as we did not detect any notable impact of the maternal past habitat on the gut microbiota of translocated juveniles. The lack of a signal of the maternal past habitat in the microbiota of offspring seems logical as these juveniles never directly experienced the past habitat of their mother and (besides a few days in captivity after being born) lived their entire lives in the habitat they were translocated to. Additionally, the reproductive strategy of bank voles could diminish the impact of maternal transmission as mothers are often pregnant with the next litter while nursing, which results in short lactation periods (approx. three weeks [63]), where the offspring receive relatively little maternal care since mothers must leave the nest for prolonged periods to forage [64]. Indeed, vertical transmission in another rodent species (wood mice, *Apodemus sylvaticus*) only resulted in a slightly higher similarity between the gut microbiota of mother–offspring pairs (approx. 22%) in comparison with unrelated pairs (approx. 19%) [14]. Besides the generally weak effect of vertical transmission in rodents, maternal transfer in mammals is also typically biased towards certain microbial taxa (e.g. *Bifidobacterium* and *Lactobacillus*, [14]), which makes it unlikely to detect more general differences in the offspring gut microbiota based upon the maternal past habitat. Surprisingly, the signal of the present habitat was also relatively weak and non-significant in the post-transfer gut microbiota of offspring. One potential reason for this could be due to age, since these juvenile rodents (less than 32 days old) heavily relied on mother milk during the majority of the exposure time in the present habitat and perhaps had not yet developed a fully mature gut microbiota community. Following the same individuals throughout their lives could provide more information on the gut microbiota of translocated juveniles after they reach adulthood, but this practice is quite challenging in natural, open systems where recapture rates are low [65].

### (c) Practical implications

Despite the ongoing conservation efforts and human-assisted wildlife translocations between different environments (e.g. reintroductions to the wild after captivity [66,67]), our understanding of the gut microbiota *flexibility* and *resistance* in wild animals remains limited [68]. Our data show that the gut microbiota of conspecifics inhabiting different habitats (including areas with different levels of human disturbance such as urban and rural forests) can have inherently different properties that can shape the gut microbiota communities even after the host has moved to a new location. Long-lasting environmental effects rather than maternal effects appear to be the driving force behind this observation, which has important practical implications for ongoing conservation programmes [30,69,70] since the gut microbiota *resistance* could prevent the host organism from using novel resources and thus potentially hinder the adaptive capacity typically offered by the gut microbiota. Therefore, we suggest that translocating very young individuals with a minimum exposure time to captivity or other previous habitats (with their mothers) or pregnant females could possibly lower the degree of *resistance* in the gut microbiota communities of these young/newborn individuals as they develop with the newly translocated habitat as their first encountered environment. Indeed, reducing time spent in captivity before translocating young captive individuals into the wild has already been proposed as a means to promote translocation success (e.g. giant pandas [18]). Importantly, studying the efficacy of faecal microbiota transplants in translocations should also be explored to improve the success rate of translocating adult individuals. One interesting and unexplored aspect in our study was the potential for functional redundancy, which perhaps gives us an inferior approximation of the adaptative potential of the bank vole gut microbiota. That said, we were unable to test this hypothesis in our dataset owing to the technical limitations imposed by marker gene sequencing. Experimental studies using both long read sequencing and shotgun metagenomics are needed to improve taxonomic resolution (in this study many ASVs could only be assigned to genus/family level) and quantify functional profiles of the bank vole gut microbiota. This information, together with long-term survival and fitness data, can help to further elucidate the role of the gut microbes in facilitating host adaptation to a change in the environment through potential differences in disease susceptibility [71,72], energy uptake from food [73], detoxification of plant compounds [74], stress physiology [55] and/or survival rate [75,76], as reported in various animal species.

## Data Availability

The raw sequences and associated metadata have been deposited to the National Center for Biotechnology Information (NCBI) under accession no. PRJNA933136 (https://www.ncbi.nlm.nih.gov/bioproject/PRJNA933136/). The R code (accessible at https://github.com/TScholier/rt-voles-workflow) and the input files are available to download at Zenodo [77]. Supplementary material is available online [78].
